# Myostatin Research: From Molecular Understanding to Clinical Translation for Musculoskeletal and Metabolic Disorders

**DOI:** 10.3390/ijms27093836

**Published:** 2026-04-25

**Authors:** Chongguang Lei, Hewen Jiang, Xin Yang, Shijian Ding, Yuanyuan Yu, Zongkang Zhang, Luyao Wang, Chong Gao, Aiping Lyu, Ling Qin, Ge Zhang, Bao-Ting Zhang

**Affiliations:** 1 School of Chinese Medicine, The Chinese University of Hong Kong, Hong Kong, China; 1155135910@link.cuhk.edu.hk (C.L.); hewenjiang@link.cuhk.edu.hk (H.J.); maxzhangzk@cuhk.edu.hk (Z.Z.); 2 Guangdong-Hong Kong Macao Greater Bay Area International Research Platform for Aptamer-Based Translational Medicine and Drug Discovery, Hong Kong, China; yangxin9613@gmail.com (X.Y.); shijian.ding@hotmail.com (S.D.); yuyuanyuan@hkbu.edu.hk (Y.Y.); luyaowang@hkbu.edu.hk (L.W.); aipinglu@hkbu.edu.hk (A.L.); 3 Law Sau Fai Institute for Advancing Translational Medicine in Bone & Joint Diseases, School of Chinese Medicine, Hong Kong Baptist University, Hong Kong, China; 4 Institute of Integrated Bioinformedicine and Translational Science, School of Chinese Medicine, Hong Kong Baptist University, Hong Kong, China; 5 School of Basic Medical Science, Guangzhou University of Chinese Medicine, Guangzhou 510405, China; chandergao@hotmail.com; 6 Musculoskeletal Research Laboratory of the Department of Orthopaedics & Traumatology, The Chinese University of Hong Kong, Hong Kong, China; lingqin@cuhk.edu.hk; 7 Innovative Orthopaedic Biomaterial and Drug Translational Research Laboratory, Li Ka Shing Institute of Health Sciences, Faculty of Medicine, The Chinese University of Hong Kong, Hong Kong, China

**Keywords:** myostatin, muscle atrophy, metabolic function, musculoskeletal disorders, pro-myostatin

## Abstract

Myostatin (Mstn), a well-characterized member of the transforming growth factor-β (TGF-β) superfamily, serves as a key negative regulator of skeletal muscle mass. Its overactivation is closely associated with the pathogenesis of various musculoskeletal and metabolic disorders. Over the past decades, inhibiting Mstn has emerged as a promising therapeutic strategy to promote muscle growth. A range of Mstn-targeted inhibitors has been developed, yielding encouraging preclinical and clinical outcomes. These include small molecules, monoclonal antibodies, peptibodies, and gene therapy-based approaches. This review summarizes the biological structure and function of Mstn, provides a comprehensive overview of recent advances in Mstn-targeted therapeutics, and offers critical insights into future directions for drug development and clinical translation.

## 1. Introduction

In 1997, McPherron et al. first identified the myostatin (Mstn) gene in mice using positional cloning. Targeted disruption of Mstn resulted in a marked increase in skeletal muscle mass, driven by both hyperplasia (increased fiber number) and hypertrophy (enlarged fiber cross-sectional area). These seminal findings established Mstn as a critical negative regulator of myogenesis and underscored its essential role in controlling muscle development [1].

The function of Mstn as a conserved negative regulator of muscle mass has since been validated across diverse vertebrate species, including multiple mammalian and teleost models. In cattle, McPherron et al. identified two distinct natural mutations linked to enhanced muscularity: an 11-nucleotide frameshift deletion in Belgian Blue cattle and a C313Y missense mutation in the Piedmontese breed. Both mutations disrupt Mstn activity, leading to profound skeletal muscle hypertrophy accompanied by reduced intramuscular fat and connective tissue [1]. Similarly, Mosher et al. characterized a 2 bp deletion in the third exon of the whippet Mstn gene, which introduces a premature stop codon. This mutation exhibits a gene-dosage effect: homozygous animals display the “bully” phenotype, marked by extreme hypertrophy and increased body mass, whereas heterozygotes show intermediate muscularity and enhanced athletic performance without the excessive bulk seen in homozygotes [2]. Beyond mammals, loss-of-function mutations or gene edits in sheep result in a 20–30% increase in muscle mass and accelerated growth. In fish, the presence of two paralogs, Mstna and Mstnb, adds further complexity. Mstna serves as the primary regulator of skeletal muscle growth in teleosts; its deletion induces a double-muscling phenotype in species such as medaka and yellow catfish. In contrast, the effects of Mstnb are species-specific: Mstnb-null Nile tilapia exhibit up to 50% greater body weight than wild-type controls, whereas Mstnb knockout in yellow catfish fails to increase muscle mass and instead leads to skeletal abnormalities [3,4].

In humans, rare polymorphisms in the Mstn gene are associated with notable muscle phenotypes. The A55T (c.164C>T) and K153R (c.458A>G) variants, for instance, have been linked to “supermuscle syndrome,” characterized by significant muscular hypertrophy and increased strength without apparent pathology. Mechanistically, the A55T mutation disrupts protein conformation and stability, reducing Mstn activity, while the K153R mutation within the mature peptide region impairs receptor-binding affinity. This attenuated signaling relieves Mstn-mediated growth suppression and correlates with enhanced muscle performance in elite strength athletes [5,6]. Further validation comes from the c.844_846delTTC deletion, which disrupts the cysteine knot critical for dimerization, leading to severe hypertrophy. A homozygous child carrying this mutation exhibited 50% greater muscle mass and grip strength compared to controls. The K153R polymorphism, by contrast, confers a milder phenotype associated with increased muscle thickness and explosive power, though its effects appear modulated by ethnicity and sex, and its relationship with endurance performance remains inconclusive [7].

This review is structured around a unifying “Activation–Systemic Crosstalk” framework. We posit that the precise molecular regulation of the latent-to-active transition serves as the primary determinant of Mstn bioavailability, while the divergence of downstream signaling dictates specific pathological outcomes, ranging from muscle wasting to metabolic dysregulation. By integrating these molecular mechanisms with clinical outcomes, we aim to provide a theoretical basis for next-generation therapeutic strategies that move beyond simple pathway inhibition to context-specific modulation.

## 2. Mstn Biology

### 2.1. Sequence and Structure

#### 2.1.1. Gene Mapping and Sequence Conservation

The Mstn gene is highly conserved, which has more than 85% sequence homology from teleosts to humans. The bioactive C-terminal mature peptide domain, which contains the seven conserved cysteines characteristic of the TGF-β superfamily, exhibits 100% amino acid identity between humans, mice, rats, pigs, chickens and turkeys. Baboon, bovine and ovine Mstn differ by only 1–3 amino acids in this region, and zebrafish shares 88% identity with humans here, underscoring the gene’s essential conserved role in vertebrates [1]. In humans, the compact three-exon structure on chromosome 2q32.2 contributes to this conservation. In humans, its compact three-exon structure on chromosome 2q32.2 contributes to this conservation [8]. Nonetheless, loss-of-function mutations disrupt protein activity and cause hypermuscularity. Notable examples include the nt821 (del11) deletion in Mstn exon 3 of Belgian Blue cattle and IVS1+5G>A splice-site mutation in Mstn intron 1 associated with human supermuscle disease [1].

#### 2.1.2. Protein Structure and Activation Process

Mstn is a critical member of the TGF-β superfamily, predominantly expressed in skeletal muscle, with lower levels detected in the heart and adipose tissue [9]. Further research has identified Mstn expression in mammary glands, spleen, lymphocytes, placenta, and even in human uterine gland muscle tissue [10,11,12,13]. Multiple Mstn isoforms exhibit differential tissue expression profiles in vivo, primarily comprising the inactive latent complex within the extracellular matrix of skeletal muscle and the active mature dimer in the serum. The circulating Mstn concentrations in healthy adults typically range from 4 to 12 ng/mL [14]. The systemic Mstn reservoir undergoes dynamic regulations, with acute exercise resulting in a transient reduction in serum Mstn levels. The biological activity [15] of Mstn is not constitutive but is tightly regulated via a multistep process involving its synthesis, secretion, and subsequent activation [16]. Analogous to other transforming growth factor beta ligands, such as activin A and GDF11, Mstn is synthesized initially as an inactive precursor (propeptide). The propeptide requires precise proteolytic cleavage and conformational modifications to transition from a latent state to a biologically active mature form. The regulatory mechanism governing latency and activation ensures that Mstn signaling occurs in a spatiotemporally restricted manner to prevent aberrant pathway activation [17]. Such a comprehensive characterization of the structure of Mstn and its specific activation cascade is essential for understanding its regulatory function [18].

Following Mstn production by skeletal muscle cells as an inactive precursor, the protein undergoes conformational alterations to form a latent complex [19]. Conserved cysteine residues within the growth factor domains form intermolecular disulfide bonds, which facilitate the formation of pro-Mstn homodimers characterized by an open V-shaped domain-swapped structure [20]. Structurally, the pro-domain arm of each monomer engages the opposing growth factor domain, stabilized by extensive hydrophobic and hydrogen bonding networks. Critically, the alpha 1 and alpha 2 helices of the forearm domain occlude the binding sites for type I and type II receptors. This steric inhibition is further reinforced by the Arg65-Tyr111-His112 motif, which utilizes aromatic stacking and salt bridges to lock the dimer in a latent conformation [17]. Due to this high stability, the latent complex constitutes the predominant extracellular isoform, accounting for over 70% of circulating Mstn [21].

**Figure 1:**
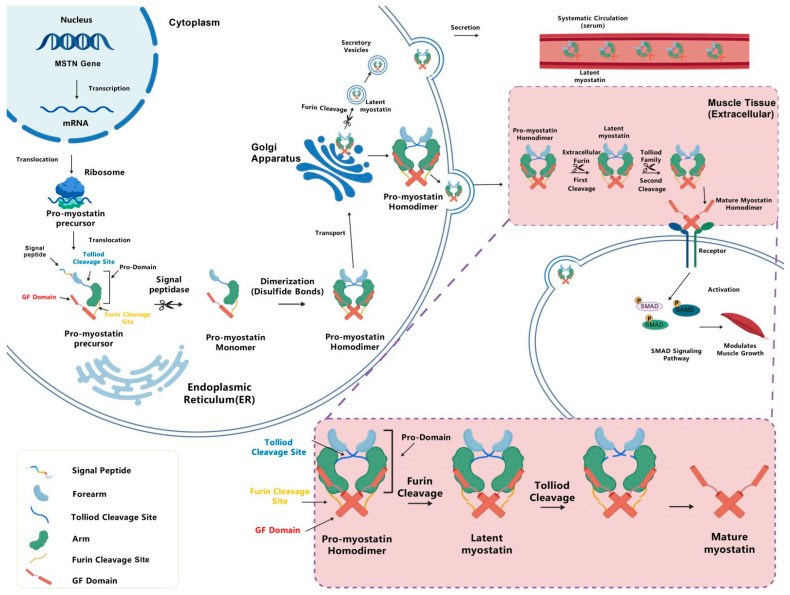
**Schematic diagram of the sequential secretion and activation of pro-Mstn.** Pro-Mstn is generated after transcription and translation of the Mstn gene, and its signal peptide is removed upon entry into the endoplasmic reticulum, after which pro-Mstn monomers form dimers. A small portion of pro-Mstn is cleaved by intracellular Furin in the Golgi apparatus to form latent Mstn for secretion into the circulation, while the majority is directly secreted into the extracellular space of muscle tissues. Extracellular pro-Mstn is sequentially processed by Furin and Tolloid family proteases to form mature Mstn dimers, which bind to type I (ALK4/5/7) and type II (ActRIIA/B) receptors on the cell surface to activate the SMAD signaling pathway and modulate muscle growth.

The activation of latent pro-Mstn involves sequential proteolytic cleavage that progressively relieves its structural constraints. The initial step involves cleavage by Furin protease, which recognizes the conserved “R-X-K/R-R” motif (Arg^263^–Arg^266^) at the junction of the pro-domain and the growth factor (GF) domain, thereafter transforming the covalently linked pro-Mstn dimer into a non-covalent latent complex [22]. Due to the occlusion of receptor-binding sites by non-covalent interactions within the domain-swapped conformation, the latent complex remains in a “low-activity latent state”, exhibiting only about 1% of the biological activity observed for the mature GF dimer [21]. Subsequent cleavage by tolloid family proteases (BMP-1 and TLL-1/2) at the Arg98/Asp99 site dissociates the pro-domain, thereby exposing the growth factor dimer. This release enables high-affinity binding to type I/II receptors, triggering the Smad2/3 signaling cascade that suppresses myogenesis (Figure 1).

### 2.2. Mstn Gene and Protein Regulation

Mstn expression is regulated by a complex network of multiple signaling pathways, involving the integration of transcriptional, post-translational, and environmental regulatory factors.

Mstn transcription is tightly regulated by a convergence of inflammatory, metabolic, and myogenic inputs. Pro-inflammatory cytokines, such as TNF-α and IL-6, along with metabolic agents like metformin, upregulate Mstn via the NF-κB and AMPK-FoxO3a-HDAC6 signaling pathways, respectively, which contribute to muscle atrophy. At the promoter level, this induction is mediated by the direct binding of activators such as FoxO3a, which compete with the myogenic repressor MyoD to dictate transcriptional output [23].

The post-translational regulation of Mstn activity hinges on sequential proteolytic processing. Accumulating evidence has confirmed that altered Furin expression directly modulates the yield of Mstn latent complex. Specifically, an increase in Furin expression significantly augments complex yield, while its suppression impairs processing of the Mstn precursor [5]. Following muscle injury, the upregulation of bone morphogenetic protein-1 (BMP-1) proteases triggers the secondary activation step. This proteolytic cleavage releases the bioactive mature Mstn dimer, which plays a vital role in limiting excessive muscle regeneration and maintaining tissue homeostasis [24].

Rather than functioning as isolated molecules, endogenous Mstn inhibitors operate as a dynamic, multi-tiered buffering system that spatially and temporally governs Mstn bioavailability. The first tier of this framework acts at the level of the extracellular matrix (ECM) to prevent premature activation. Growth and differentiation factor-associated serum proteins 1 and 2 (GASP-1/2) specifically recognize and bind to the pro-domain of latent Mstn complexes. By occupying these pro-domain regions, GASP proteins sterically shield Mstn from cleavage by Tolloid family proteases (e.g., BMP-1), thereby maintaining Mstn in a dormant state within the local ECM microenvironment and preventing uncontrolled localized activation [25,26]. When tissue homeostasis is disrupted—such as during muscle injury where Tolloid proteases are highly upregulated—the first tier is overwhelmed, leading to the release of mature Mstn dimers. The system then shifts to a second tier of defense: rapid, high-affinity systemic neutralization. Follistatin (FST) acts as an efficient “emergency brake” in this dynamic framework. With a remarkably low half-maximal inhibitory concentration (0.36 nM), FST directly sequesters liberated mature Mstn dimers before they can engage ActRIIB receptors on the cell surface [27,28]. Together, this dual-layered framework, comprising GASP-mediated spatial sequestration of the latent complex and FST-mediated acute neutralization of the mature dimer, creates a robust negative-feedback loop. This dynamic interplay ensures that Mstn signaling is tightly restricted to precise pathophysiological windows (e.g., limiting excessive regeneration post-injury), preventing aberrant pathway activation while preserving baseline tissue homeostasis.

Environmental stimuli such as exercise and muscle disuse function as macro-level regulators that dynamically rewire the entire Mstn signaling network. Mechanical loading (exercise) integrates into this network through dual mechanisms: it suppresses Mstn bioavailability at the epigenetic and translational levels (e.g., endurance exercise remodels Mstn mRNA stability via Ythdf1-mediated m6A modifications, and resistance exercise downregulates Mstn transcription while upregulating myogenin [28]) https://ouci.dntb.gov.ua/en/works/lDdmDAmM/ (accessed on 21 April 2026).

In addition, muscle disuse (e.g., bed rest) integrates into the network by actively reshaping the muscle microenvironment to sensitize Mstn signaling. Disuse triggers chronic low-grade inflammation and adipose tissue infiltration, leading to the elevation of pro-inflammatory cytokines (TNF-α and IL-6). These cytokines do not act in isolation; they hijack the Mstn network by synergizing with NF-κB and FoxO3a to amplify Mstn transcription [23] https://ouci.dntb.gov.ua/en/works/lDdmDAmM/ (accessed on 21 April 2026). Furthermore, disuse-induced metabolic dysfunction may suppress the expression of endogenous buffers like Follistatin. Thus, disuse drives a pathogenic feed-forward loop where an inflamed niche not only produces more Mstn but also lowers the activation threshold for its downstream catabolic machinery (Smad2/3-FoxO-ubiquitin ligases), leading to the observed 12% increase in circulating Mstn and progressive atrophy [29].

### 2.3. Core Signaling Pathways of Mstn

#### 2.3.1. Classical Signaling Pathway

The canonical signaling cascade is triggered by high-affinity binding of the mature Mstn dimer to ActRIIB (Kd = 1–2 nM), inducing a conformational change in ActRIIB [30]. The type I receptors (ALK4/5) are then recruited and transphosphorylated, forming an active signaling complex. This active complex then phosphorylates Smad2/3 at C-terminal SSXS motifs, facilitating their association with Smad4 and subsequent nuclear translocation. In the nucleus, the Smad complex orchestrates transcriptional reprogramming by repressing myogenic factors (MyoD and Myogenin) while upregulating E3 ubiquitin ligases (Atrogin-1 and MuRF-1). This regulatory mechanism ultimately suppresses protein synthesis and accelerates proteolysis [31].

#### 2.3.2. Non-Classical Signaling Pathways

In addition to the canonical Smad-dependent pathway, Mstn modulates muscle metabolism through several non-Smad signaling cascades. Mstn is shown to suppress Akt phosphorylation at Ser^473^, thereby inhibiting mTORC1 activity and ultimately reducing muscle protein synthesis [32,33]. In mouse models of Duchenne muscular dystrophy (DMD), Mstn inhibition leads to a 40–50% restoration of Akt/mTOR signaling, which contributes to partial amelioration of muscle atrophy [34]. Furthermore, Mstn activates the MAPK pathway, particularly through the TAK1–MKK3/6–p38 MAPK axis, which impedes myogenic differentiation [35]. Consistently, treatment with Mstn in myocytes results in a more than two-fold increase in phosphorylated p38 level and a 30% decrease in the myotube fusion index, indicating impaired myogenic differentiation [36].

The canonical and non-canonical Mstn pathways do not operate in isolation but form a highly integrated regulatory network centered on signal convergence. At the core of this systems-level crosstalk is the synergistic interaction between Smad complexes and FoxO transcription factors. While Mstn-induced Akt suppression (Ser^473^) inhibits mTORC1 to arrest protein synthesis, it concurrently acts as a permissive gate for FoxO nuclear translocation. In the nucleus, FoxO proteins physically associate with the Smad2/3-Smad4 complex, forming a synergistic transcriptional hub that drives supra-additive expression of E3 ubiquitin ligases (Atrogin-1 and MuRF-1) [37,38]. Furthermore, TAK1 serves as a critical bifurcating node downstream of ActRIIB; it simultaneously activates the p38 MAPK axis to impede myogenic differentiation (via MyoD suppression) and modulates Smad linker-region phosphorylation to sustain transcriptional output. This interconnected “Hub-and-Spoke” model, where Akt inhibition enables FoxO-Smad catabolic transcription, TAK1 bridges differentiation arrest and proteolysis, and mTORC1 suppression prevents compensatory anabolism, explains why isolated blockade of Smad signaling often fails to fully rescue muscle quality in complex pathophysiological environments like DMD and sarcopenia.

### 2.4. Activity Assessment of Pro-Mstn and Mature Mstn

#### 2.4.1. Cell Proliferation

Cell proliferation assays serve as fundamental tools for evaluating the biological activity of pro-Mstn and its other forms by directly measuring their functional impact on muscle cell growth. The C2C12 mouse myoblast line remains the predominant model, and diverse cell types (e.g., HEK293T, MG-63, and CHO) are utilized to assess functional impact. Quantification strategies include direct enumeration, high-throughput metabolic viability assays (MTT and CCK-8), and DNA synthesis analysis via [^3^H]thymidine incorporation or EdU/BrdU labeling. These methods have confirmed the capacity of Mstn to induce G1/S phase arrest. Notably, treatment with 25 nM recombinant Mstn suppresses DNA and total protein synthesis in C2C12 myoblasts by 68% and 37%, respectively, in a dose-dependent manner [39].

#### 2.4.2. Dual Luciferase Reporter Assay System

The dual luciferase reporter assay provides a sensitive and quantitative method for assessing Mstn activity by measuring downstream Smad2/3-dependent transcriptional activation in cells [22]. The successful application of the dual luciferase reporter assay is demonstrated across multiple cellular contexts. In C2C12 mouse myoblasts, studies identify microRNA-mediated regulation of Mstn mRNA expression, particularly via miR-27a targeting the Mstn 3′UTR [40]. Given that short RNAs are inherently prone to degradation by serum nucleases, asymmetric terminal modification might significantly enhance their serum stability [41]. Additionally, CHO-K1 cells are employed for the functional characterization of recombinant Mstn inhibitors such as FST [21].

Dual luciferase reporter assays combined with alanine-scanning mutagenesis in HEK293T cells elucidate the structural determinants of pro-Mstn activation. Specifically, mutations at residues Y94, D95, and V96 (proximal to the Furin cleavage site) significantly compromise proteolytic cleavage, suppressing luciferase activity by >80% relative to wild-type. Conversely, the D103A substitution augments Smad-responsive transcriptional activity by approximately 40%, implicating Asp103 in the stabilization of the latent conformation [42]. Moreover, Szláma et al. demonstrate that variant K153R accelerates Furin-mediated activation of pro-Mstn and significantly enhances Smad-responsive transcriptional output [5]. Similarly, on the basis of the dual luciferase system, Takayama et al. quantitatively measure the inhibitory potency of pro-domain-derived peptides, identifying the peptide fragment (residues 65 to 82) from the mouse pro-domain as the minimum effective unit capable of potently suppressing human Mstn activity [43].

#### 2.4.3. Smad2/3 Phosphorylation Detection

**Figure 2:**
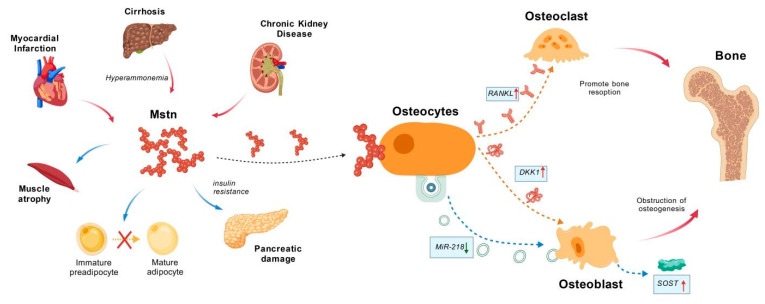
**The role of Mstn in multiple diseases.** In patients with myocardial infarction, cirrhosis or chronic kidney disease, serum Mstn levels are higher than those of healthy subjects, which contributes to muscle atrophy, adipogenic differentiation of preadipocytes and insulin resistance. Furthermore, Mstn suppresses the level of microRNA-218 (miR-218) in osteocyte-derived exosomes, which leads to increased sclerostin expression in osteoblasts. Moreover, Mstn directly acts on osteocytes and upregulates bone formation inhibitors and bone resorption promoters, subsequently inhibiting the Wnt/β-catenin signaling pathway and resulting in bone loss.

Western blot analysis of Smad2/3 phosphorylation is extensively utilized as a fundamental method to evaluate the bioactivity of mature Mstn by detecting its downstream signaling events. The critical role of Smad3 is demonstrated in mechanistic studies where Smad3-deficient mouse models are protected from Mstn-induced muscle atrophy [44]. Clinical observations further establish that the phosphorylated Smad2/3 (pSmad2/3) levels detected by this assay serve as a reliable molecular biomarker, exemplified by their elevation in muscle biopsies from patients with sporadic inclusion body myositis [45]. Furthermore, treatment with bimagrumab, a monoclonal antibody that blocks the activin type II receptors to inhibit signaling by both Mstn and activins, leads to decreased pSmad2/3 levels in skeletal muscle. This inhibition of the pathway is associated with increased muscle mass and subsequent improvements in muscle strength and physical function in patients [46]. Additionally, research reveals alternative phosphorylation mechanisms, such as JNK-mediated phosphorylation of Smad2 at linker regions during resistance exercise, which fine-tunes pathway activity in a context-dependent manner (Figure 2) [47].

## 3. Physiological and Pathophysiological Roles of Mstn

### 3.1. Physiological Roles of Mstn

Mammalian skeletal muscle fibers are classified as glycolytic or oxidative based on their metabolic profiles, corresponding to fast type IIB and slow type I/IIA MHC isoforms in mice, respectively [48]. Beyond its role in fiber identity, myostatin plays a robust role in protecting undifferentiated myoblasts from apoptosis and has concentration-dependent effects on the proliferation and activity of C2C12 cells; it inhibits their proliferation at high concentrations (80–400 nM) [41,49] and promotes their proliferation at low concentrations (2–20 nM). Mstn is also a key regulator in the process of muscle fiber transformation. Mstn-KO mice display a shift from slow oxidative (type I/IIA) to fast glycolytic (type IIB) fibers, resulting in an overall faster, more glycolytic, and fatigable muscle phenotype [50]. Muscle fiber transformation is driven by the dual role of Mstn in regulating fiber-type-specific pathways, promoting the slow-fiber MEF2C/calcineurin pathway while inhibiting the fast-fiber MyoD pathway. Accordingly, *Mstn*-KO mice show reduced MEF2C activity and calcineurin expression (down 50%) alongside elevated MyoD. This mechanism is further supported by Mstn-mutant cattle, which exhibit downregulation of the MEF2C target MLC1f, and by in vitro studies where Mstn activates the MLC1f promoter through MEF2C and MyoD [51]. However, it is critical to note that this fiber-type switching appears to be a consequence of developmental processes, as inhibiting Mstn in adult animals does not induce a similar fast-glycolytic transformation [52].

### 3.2. Pathophysiological Roles of Mstn

#### 3.2.1. Age-Related Sarcopenia

Sarcopenia, defined by the progressive involution of skeletal muscle mass and strength in older adults, exhibits a robust correlation with elevated Mstn signaling. Epidemiological analyses indicate a 30–50% increase in serum Mstn levels among individuals over 60 years of age, an elevation that exhibits a strong inverse association with muscle mass [53]. This age-dependent upregulation is multifactorial, driven by chronic low-grade inflammation, mitochondrial dysfunction, and impaired satellite cell activation [51]. Consistently, Yarasheski et al. demonstrated that serum Mstn-immunoreactive protein levels are highest in women aged 76–92 years, with fat-free mass (FFM) showing an inverse correlation with circulating Mstn [54]. In mouse models, Mstn-null mice exhibit remarkable preservation of muscle mass into senescence in contrast to wild-type controls. Furthermore, the regenerative capacity of skeletal muscle in aged Mstn-deficient mice is significantly superior to that of wild-type cohorts [55].

Mechanistically, the metabolic imbalance in sarcopenia is driven by dual pathogenesis. On the one hand, the anabolic drive is suppressed, as evidenced by a 40% reduction in phosphorylated Akt specifically within aged muscle [56]. On the other hand, proteolysis is concurrently accelerated. This latter process is mediated by the cooperative activation of FoxO transcription factors via Smad3 and p38 MAPK signaling, which upregulates the E3 ubiquitin ligases Atrogin-1 and MuRF1 to drive the progressive protein breakdown characteristic of sarcopenic tissue [37,57]. Beyond proteolytic regulation, Mstn exacerbates sarcopenia through detrimental effects on mitochondrial function [38]. Mstn signaling impairs the AMPK-PGC-1α pathway, thereby attenuating mitochondrial biogenesis and compromising the capacity of muscle cells to oxidize lipids for energy [58].

Although preclinical models suggest robust anabolic potential, clinical trials frequently fail to demonstrate tangible functional benefits. For instance, in a randomized clinical trial evaluating bimagrumab (a monoclonal antibody targeting ActRIIA/B receptors) in sarcopenic older adults, subjects receiving concurrent nutritional support and light exercise show no significant difference compared to the placebo group, as physical function improves in both groups [59]. Similarly, a Phase 2 trial of bimagrumab (a monoclonal antibody targeting ActRIIA/B receptors) in frail older adults establishes that while 24 weeks of treatment significantly increased appendicular lean mass, improvements in primary functional endpoints, such as gait speed, are not consistently significant. Consequently, despite the demonstrated ability of these agents to augment muscle mass, their clinical development is discontinued. This recurrent failure implies that simply inhibiting Mstn may be insufficient to address the underlying metabolic defects, such as mitochondrial dysfunction, that compromise muscle quality in aging [60].

This recurrent discrepancy between morphological and functional outcomes can be explained by several key mechanistic factors. First, fiber-type switching plays a critical role; as noted in Section 3.1, Mstn inhibition promotes a shift from slow-twitch oxidative fibers (Type I/IIA) to fast-twitch glycolytic fibers (Type IIB). While this shift contributes to hypertrophy, it results in a muscle phenotype that is more fatigable and metabolically less efficient, thereby limiting improvements in physical endurance and strength. Second, signaling redundancy may undermine therapeutic efficacy; blockade of the ActRIIB receptor can trigger compensatory upregulation of related ligands, such as activin A, which sustain catabolic signaling and suppress anabolic pathways via similar receptors. Finally, simply increasing muscle fiber size does not rectify the underlying structural defects of the aged muscle microenvironment, such as fibrosis and fatty infiltration, which mechanically impair force transmission. Therefore, this “functional failure” implies that simply inhibiting Mstn may be insufficient to address the comprehensive metabolic and structural defects that compromise muscle quality in aging [61].

#### 3.2.2. The Role of Mstn in Bone Diseases

Mstn functions as a critical regulator in bone-related diseases and its dysregulation is increasingly implicated in the pathogenesis of diverse skeletal disorders, including osteoarthritis (OA), metastatic bone disease, fracture, and metabolic bone disease. Patients with OA exhibit significantly higher serum Mstn concentrations than healthy controls [16,61]. Similarly, in the context of breast cancer bone metastases, Mstn expression is notably enriched within tumor cells at the bone-tumor interface of patient-derived lesions, suggesting that Mstn has a potential role in facilitating the osteolytic microenvironment [62]. In an osteoporotic fracture model utilizing sarcopenic SAMP8 mice, Mstn expression is significantly upregulated in both the fracture callus and the adjacent biceps femoris muscle compared to non-sarcopenic controls, highlighting the detrimental role of Mstn in the impaired fracture healing associated with sarcopenia. In vitro co-culture assays confirm that exposure to aged myofibers suppresses the expression of osteogenic markers (ALP and Runx2) in MC3T3-E1 cells, which is reversed by Mstn inhibition. In diet-induced obese rats, it is also reported that intervention with a polyclonal anti-Mstn antibody can reverse trabecular bone loss and microarchitectural deterioration, suggesting that inhibiting Mstn signaling offers a promising avenue for ameliorating obesity-related metabolic bone disease [63].

As a pivotal mediator of the muscle-bone axis, Mstn is reported to directly target osteocytes, the master mechanosensory cell of bone, to upregulate the expression of sclerostin (encoded by the SOST gene), RANKL, and Dickkopf-1 (Dkk-1). Sclerostin subsequently antagonizes the canonical Wnt/β-catenin signaling pathway in osteoblasts, thereby suppressing osteogenesis. Dysregulation of the Mstn-Sclerostin-Wnt/β-catenin axis is a significant contributor to bone loss in conditions such as sarcopenia and disuse osteoporosis [64]. Furthermore, Mstn suppresses the abundance of microRNA-218 (miR-218) in osteocyte-derived exosomes, and when these exosomes are internalized by osteoblasts, the reduced delivery of miR-218 leads to unrestrained sclerostin expression, further inhibiting Wnt/β-catenin signaling and impairing osteoblast differentiation [65].

Crucially, the osteogenic benefits of Mstn inhibition are strictly dependent on mechanical loading. While Mstn deficiency typically drives bone accretion, hindlimb unloading abolishes this effect, instead favoring bone marrow adipogenesis. This reversal suggests that Mstn regulates the mechanosensitivity of mesenchymal stem cells, likely by gating the loading-induced expression of osteogenic factors like BMP-2 and IGF-1 [64]. Recently, a dual-action LYTAC approach leveraging bone–liver crosstalk achieved bone-specific accumulation and protein degradation [66], highlighting the potential of nucleic acid-based platforms for bone disorders [67,68].

#### 3.2.3. Duchenne Muscular Dystrophy (DMD)

DMD is a severe X-linked recessive disorder induced by mutations in the dystrophin gene, resulting in the absence of functional dystrophin protein. Dystrophin is crucial for maintaining sarcolemmal integrity during muscle contraction [69]. Dystrophin deficiency triggers repeated cycles of muscle fiber damage, inflammation, and impaired regeneration, ultimately leading to progressive muscle wasting and replacement by fibrotic and adipose tissue [70].

Interestingly, Mstn has emerged as a key regulator that exacerbates DMD progression. In DMD mouse models, it is found that persistent muscle injury significantly upregulates Mstn expression (frequently 2~3-fold higher in dystrophic muscle compared to healthy controls) [71]. The overexpression directly impairs satellite cell-mediated regeneration by arresting satellite cells in the G1 phase through upregulation of the cell-cycle inhibitor p21 and downregulation of cyclin-dependent kinase Cdk2, thereby suppressing their activation, proliferation, and self-renewal [72]. Concomitantly, Mstn downregulates key myogenic factors, such as MyoD and Myf5, further inhibiting myogenesis [72,73,74]. Moreover, in mouse models, Mstn overexpression accelerates muscle atrophy by promoting the ubiquitin–proteasome system via upregulation of E3 ligases, including MuRF1 and Atrogin-1 [37]. The suppression of regeneration and the acceleration of atrophy both contribute to the imbalance between muscle degradation and regeneration, which is a characteristic feature of DMD pathology [75]. Notably, even under conditions of severe satellite cell deficiency or dysfunction, inhibition of the Mstn/activin A pathway can still induce muscle hypertrophy by acting directly on myofibers, suggesting that therapeutic targeting of this pathway may retain efficacy in DMD and other conditions where satellite cell function is compromised [76].

Based on these mechanisms, Mstn inhibitors are explored for DMD treatment. In a Phase 2 randomized controlled trial involving patients with DMD, the effect of domagrozumab (a monoclonal antibody targeting Mstn) is evaluated. Although the treatment leads to a modest increase in muscle mass (approximately 3%), it fails to demonstrate significant improvements in functional outcomes (e.g., 45 m walk time or upper limb strength) [49]. It suggests that Mstn is a component of the complex pathological network in DMD, where other factors including chronic inflammation, oxidative stress, and fibrosis might also irreversibly impair the muscle microenvironment [77].

Mechanistically, the discordance between muscle hypertrophy and functional improvement in DMD can be attributed to the fact that the qualitative integrity of muscle tissue is compromised by extensive fibrosis and fatty infiltration. While Mstn inhibition induces hypertrophy of residual myofibers, it does not concurrently reverse the pathological remodeling of the extracellular matrix (ECM) or adipose replacement, thereby decoupling muscle cross-sectional area from effective force generation.

#### 3.2.4. Spinal Muscular Atrophy (SMA)

SMA is a severe neuromuscular disorder resulting from mutations in the SMN1 gene, and is characterized by survival motor neuron (SMN) protein deficiency and progressive degeneration of spinal motor neurons. This degenerative process predominantly targets proximal musculature, manifesting clinically as hypotonia and profound atrophy. The disease spectrum is heterogeneous, ranging from acute infantile-onset forms complicated by respiratory and bulbar compromise to milder, late-onset phenotypes [78].

The role of Mstn in SMA has been extensively studied. Unexpectedly, both clinical cohorts and murine models exhibit reduced serum Mstn levels. This apparent paradox, wherein blocking Mstn yields clinical benefits despite low systemic levels, is resolved by distinguishing between circulating reservoirs and local tissue activity. Serum Mstn predominantly reflects the overflow of inactive latent complexes and does not accurately capture the dynamic, autocrine or paracrine activation occurring within the specific muscle microenvironment. In pathological states, local proteolytic activity (e.g., upregulated Tolloid proteases in the extracellular matrix) can drive the localized release of mature Mstn independent of serum concentrations. Therefore, therapies like apitegromab exert their efficacy by intercepting this local ECM-bound latent pool, irrespective of systemic serum levels [79].

Therapeutic blockade via AAV-mediated propeptide of Mstn expression yields efficacy contingent upon disease severity, while monotherapy confers negligible survival or functional benefits in severe phenotypes, it significantly augments muscle mass and motor performance in milder cases, particularly as an adjunct to SMN-restoring therapies [80]. Clinically, in a Phase 3 TOPAZ trial, apitegromab, a monoclonal antibody targeting latent Mstn, exhibits notable enhancements in motor function for children aged 2–12 with later-onset SMA types 2 or 3. Apitegromab (a monoclonal antibody targeting latent Mstn) stands out as the initial effective muscle-targeted therapy among Mstn inhibitors for SMA [81]. Additionally, taldefgrobep alfa, a recombinant ActRIIB-Fc fusion protein, is presently under assessment in the Phase 3 RESILIENT trial as a complement to SMN-upregulating treatments such as risdiplam [79].

#### 3.2.5. Limb-Girdle Muscular Dystrophy (LGMD)

Limb-Girdle Muscular Dystrophy type 1C (LGMD1C) is a dominantly inherited muscle disease caused by mutations in the CAV3 gene. The encoded protein, caveolin-3, normally binds ALK4/5 to suppress the Mstn-Smad2-p21 axis. Loss of functional caveolin-3 leads to hyperactivation of the pathway. Unchecked signaling drives muscle hypoplasia and atrophy, defining the disease phenotype. Consequently, Mstn inhibition effectively rescues muscle atrophy and function in LGMD1C models, validating the central pathogenic role of Mstn [82]. In contrast, in δ-sarcoglycan-deficient mice (LGMD2F), the benefit of Mstn inhibition is highly dependent on timing [83]. Early intervention improves muscle mass and regeneration, whereas late treatment fails to ameliorate pathology and can even exacerbate fibrosis [84].

#### 3.2.6. Cancer Cachexia

Cachexia is a debilitating, multifactorial metabolic syndrome complicating the course of diverse chronic pathologies, including malignancy, sepsis, chronic kidney disease, cardiac failure, and acquired immune deficiency syndrome [85,86]. The hallmark of cancer cachexia is involuntary and progressive weight loss, characterized primarily by the depletion of adipose tissue and severe skeletal muscle atrophy [85]. Regarding the Mstn expression in clinical cohorts, findings remain equivocal. Mstn mRNA levels are frequently reported as unchanged [87] or even downregulated [88] in the skeletal muscle of cachectic cancer patients. However, these cross-sectional data do not preclude the possibility of a transient upregulation of Mstn during the early phases of disease progression, prior to the onset of overt muscle wasting.

Genetic ablation of Mstn effectively mitigates skeletal muscle wasting in mice engrafted with Lewis lung carcinoma (LLC) cells and in ApcMin/+ mice, an established model of colorectal cancer-associated cachexia. Notably, the absence of Mstn also impedes LLC tumor growth and reduces the size and burden of intestinal polyps in ApcMin/+ mice, thereby conferring a significant survival advantage in both models [89]. The pro-inflammatory tumor microenvironment plays a critical regulatory role in transcriptional expression of Mstn cytokines, such as TNF-α and IL-6, which induce the transcriptional upregulation of Mstn [90]. Concurrently, chronic inflammation suppresses FST expression via epigenetic silencing of the FST promoter. Consequently, hyperactive Mstn signaling drives muscle proteolysis [91].

Clinically, although anti-Mstn antibodies, such as domagrozumab, demonstrate efficacy in increasing muscle mass in patients with non-small-cell lung cancer, they frequently fail to translate into functional improvements. This dissociation between mass and function may be attributable to a compensatory upregulation of activin A, which results in incomplete blockade of the pathway [92]. In addition, agents like IMB0901, a Mstn inhibitor, have shown promise in mitigating cancer cachexia-induced atrophy by simultaneously suppressing ubiquitin-mediated proteolysis and promoting protein synthesis [93]. Aptamer-based active targeting may significantly enhance the therapeutic efficacy of inhibitors against tumors while reducing their toxic side effects [94,95]. In particular, a delivery system for aptamers could significantly enhance their penetration and inhibitory effects [96]. In this context, the development of aptamer–Mstn inhibitor conjugates could offer a promising strategy to simultaneously counteract muscle wasting and suppress tumor progression.

#### 3.2.7. Chronic Kidney Disease (CKD) Related Muscle Wasting

Under physiological conditions, the kidney acts as a primary clearance organ for circulating Mstn. Additionally, circulating Mstn concentrations are inversely correlated with renal function [97]. Beyond this systemic effect, local dysregulation of Mstn within the kidney is a key feature of kidney disease. In diabetic nephropathy (DN) patients, renal biopsies show marked upregulation of Mstn mRNA and protein levels, particularly in the tubulointerstitial compartment. The expression of both protein and mRNA levels correlates positively with interstitial fibrosis and inflammation [98]. In DN patients, Mstn expression is markedly upregulated in renal tissues. Consistently, serum Mstn concentrations in patients with end-stage renal disease exceed levels in healthy individuals by two- to three-fold. Verzola et al. report that Mstn mRNA expression in kidney biopsies from patients with CKD is 10-fold higher than in healthy subjects, and Mstn protein concentration is four-fold higher [99].

Metabolic stimuli such as high glucose and glycated albumin upregulate Mstn and ActRIIB in renal tubular cells, driving renal injury. Mechanistically, Mstn signaling triggers the phosphorylation of NF-κB and Smad2/3, thereby concurrently activating inflammatory and pro-fibrotic cascades. Critically, Mstn silencing abrogates these pathogenic responses to metabolic stress [98].

The clinical observations are substantiated by preclinical data derived from animal models. In a rat subtotal nephrectomy model, muscle Mstn mRNA levels in muscle are increased compared with the control group, which return to baseline values following two to seven days of exercise [100]. Similarly, in a 5/6 nephrectomy mouse model, mice with CKD display higher levels of IL-6, Mstn, and Atrogin-1 mRNA in the gastrocnemius muscle alongside lower Akt phosphorylation [101]. Mechanistically, the chronic low-grade inflammatory state, characterized by elevated TNF-α and IL-6 levels combined with accumulated uremic toxins such as indoxyl sulfate, drives the pathology. Cytokines promote Mstn transcription via the JAK/STAT3 pathway, while uremic toxins directly stimulate Mstn mRNA expression in muscle tissue. The elevated secretion of Mstn exacerbates muscle atrophy and clinical symptoms [102,103]. A clinical study demonstrates that Mstn is the only independent factor that predicts sarcopenia in the result of multivariate analysis, indicating that the serum Mstn level may be a promising biomarker for the early diagnosis of sarcopenia [104].

#### 3.2.8. Chronic Obstructive Pulmonary Disease (COPD)

COPD is a systemic disease disorder associated with key musculoskeletal complications, such as sarcopenia. In patients with COPD, Mstn expression is upregulated in skeletal muscle [105,106]. A study involving male patients reports a negative correlation between serum Mstn levels and skeletal muscle mass [107]. Recent histological analyses of the vastus lateralis muscle in sarcopenic patients reveal decreased markers of muscle regeneration, including Pax-7, Myf-5, MyoD, and myogenin, alongside reduced numbers of Pax-7+/Myf-5- satellite cells. Conversely, the tissue exhibits elevated markers of muscle injury and increased Mstn levels [108]. In a clinical trial involving sarcopenia patients with COPD, the Mstn inhibitor bimagrumab (a monoclonal antibody targeting ActRIIA/B receptors) increases skeletal muscle mass but failed to improve muscle function or physical performance [109]. It suggests that Mstn has the potential to serve as a biomarker for muscle atrophy in patients with COPD.

Cigarette smoke induces a catabolic program by upregulating Mstn via Erk1/2 activation. Elevated Mstn then drives atrophy by inducing E3 ligases (MuRF1, Atrogin-1), suppresses myogenesis by inhibiting satellite cells, and downregulates the myokine irisin (Fndc5) through a Smad3/PGC-1α pathway. This multi-pronged suppression of anabolic and myokine signaling accelerates muscle dysfunction [110].

Currently, resistance exercise remains a cornerstone intervention for muscle loss in patients with COPD. The intervention effectively lowers Mstn levels and improves muscle function [111].

#### 3.2.9. Cardiac Cachexia

Cardiac cachexia is a severe complication of advanced chronic heart failure, characterized by progressive skeletal muscle and fat loss that significantly increases morbidity and mortality [112,113]. A clinical study shows that plasma concentrations of Mstn are significantly increased in patients with congestive heart failure relative to healthy controls [114]. Notably, sex differences may influence this Mstn-mediated muscle-wasting process, as female patients often exhibit higher myocardial Mstn and pSmad2 levels, potentially predisposing them to more severe cachexia [115]. This cardiac-derived Mstn signaling is often amplified by systemic factors common in heart failure, such as chronic inflammation and insulin resistance, which further accelerate muscle loss [116,117].

Preclinical investigations elucidate a causal axis between cardiac stress and sarcopenia. In a rat volume-overload model, a 2.7-fold upregulation of myocardial Mstn coincides with significant skeletal muscle atrophy [118]. Heineke et al. confirmed that the myocardium drives peripheral wasting, as blocking cardiac Mstn prevented systemic elevation and muscle loss in murine heart failure. Pathological cardiac stress upregulates Mstn, contributing to local remodeling and secretion into the circulation [119].

Collectively, these data indicate that pathological cardiac stress induces a rapid upregulation of Mstn production within the myocardium. This elevated cardiac Mstn not only contributes to local myocardial remodeling and fibrosis but is also secreted into the circulation [119,120].

#### 3.2.10. Obesity

Obesity is characterized by energy imbalance and adipose tissue dysfunction. Clinical evidence demonstrates elevated Mstn protein expression in skeletal muscles of obese individuals, correlating with insulin resistance and systemic inflammation [121]. Mechanistic studies suggest that elevated Mstn may contribute to metabolic dysfunction by impairing glucose uptake in adipose tissue and skeletal muscle [122]. Genetic ablation or antibody-mediated inhibition of Mstn enhances energy expenditure and restores the thermogenic function of brown adipose tissue [123]. In addition, Mstn may also participate in central metabolic regulation through hypothalamic agouti-related peptide (AgRP) neurons. Activation of AgRP neurons suppresses sympathetic outflow to brown adipose tissue and upregulates Mstn protein expression, resulting in reduced glucose uptake in adipose tissue [124].

Therapeutically, the combination of Mstn inhibition and other therapies has already been used to counteract obesity. The combination of Mstn inhibitors and GLP-1 receptor agonists enables high-quality weight loss by prioritizing fat reduction while preserving or increasing lean body mass (LBM). While GLP-1 receptor agonists deliver robust weight loss (15–20%), 20–40% of this reduction originates from LBM, leading to decreased resting metabolic rate (RMR) and heightened post-treatment weight regain. In contrast, Mstn inhibitors could uncouple fat loss from muscle depletion. In a 48-week Phase 2 trial involving overweight or obese T2DM patients, bimagrumab (a monoclonal antibody targeting ActRIIA/B receptors) reduces fat mass by 20.5% and increases LBM by 3.6%, with 100% of the weight loss derived exclusively from adipose tissue [125]. The synergistic potential of combination therapy is further validated in the BELIEVE trial. In 507 obese participants followed for 72 weeks, semaglutide (a GLP-1 receptor agonist) plus bimagrumab (a monoclonal antibody targeting ActRIIA/B receptors) achieved a cumulative weight loss of 22.1% (surpassing the 15.7% reduction with semaglutide alone), with 92.8% of lost weight from fat and a 67% improvement in muscle preservation (LBM loss of −2.6% vs. −7.9% with semaglutide monotherapy) (https://clinicaltrials.gov/ct2/show/NCT05616013, accessed on 13 Feburary 2026). This synergy arises from complementary mechanisms where GLP-1 receptor agonists reduce energy intake via appetite suppression and delayed gastric emptying, while Mstn inhibitors remodel body composition by sustaining muscle mass (preserving RMR) and promoting white adipose tissue browning. These combined actions collectively mitigate rebound risk and avoid sarcopenic obesity.

**Figure 3:**
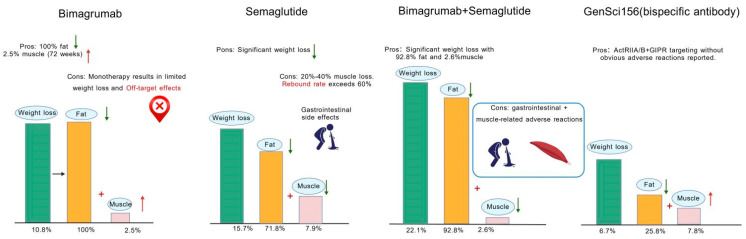
Comparative profiles of four weight-loss therapies (left to right: bimagrumab (a monoclonal antibody targeting ActRIIA/B receptors), semaglutide, bimagrumab+semaglutide, and GenSci156 bispecific antibody): (1) Bimagrumab delivers 10.8% weight loss (100% fat reduction and 2.5% muscle gain over 12 weeks), offering exclusive fat loss plus muscle gain, though limited by monotherapy’s modest weight loss and off-target effects. (2) Semaglutide induces 15.7% weight loss (71.8% fat reduction and 7.9% muscle loss) and drives significant weight loss, yet brings 20–40% muscle loss, >60% rebound, and gastrointestinal side effects. (3) The bimagrumab+semaglutide combination yields 22.1% weight loss (92.8% fat reduction and 2.6% muscle loss), which is a substantial, fat-predominant loss, though it is associated with combined gastrointestinal and muscle-related adverse reactions. (4) GenSci156 (ActRIIA/B+GIPR-targeting) results in 6.7% weight loss (25.8% fat reduction and 7.8% muscle gain) with no reported obvious adverse reactions.

Additionally, the development of bispecific antibodies to achieve better metabolic improvement while minimizing muscle loss has also garnered extensive research interest. GenSci156 is a bispecific antibody targeting both Activin II receptors (ActRIIA/B, key mediators of Mstn signaling) and glucose-dependent insulinotropic polypeptide receptor (GIPR) developed by Jinsai Pharmaceutical Co., Ltd. in Changchun, China. Its advantages lie in synergistically blocking Mstn-mediated metabolic disorders and regulating glucose metabolism, achieving fat reduction while increasing muscle mass without affecting appetite. But its long-term safety and efficacy in human trials remain to be confirmed (Figure 3) [126].

#### 3.2.11. Type 2 Diabetes Mellitus (T2DM)

Clinical observations indicate that Mstn mRNA expression is upregulated specifically within the skeletal muscle of patients with T2DM. The microarray gene expression analysis demonstrates elevated Mstn mRNA levels in skeletal muscle biopsies from patients with T2DM [127]. In larger cohorts, Mstn levels in the plasma of healthy young men dramatically drop 24 h after exercise compared to pre-exercise and are positively correlated with plasma Interleukin-6 (IL-6) [128]. Consistent with this, aerobic exercise interventions in insulin-resistant middle-aged men have been shown to attenuate both muscle and plasma Mstn protein levels, as well as improve insulin sensitivity [129].

In *Mstn*-KO mice, insulin sensitivity is markedly improved compared to wild-type controls. McPherron et al. establish that *Mstn*-KO mice have a significant reduction in fat accumulation with increasing age [130]. In addition, Mstn mutation can partially suppress both fat accumulation and the development of hyperglycemia in genetic mouse models of obesity [130]. Subsequent investigations utilizing diet-induced obesity models substantiate these metabolic benefits. Specifically, Zhao et al. and Guo et al. demonstrate that genetic ablation or muscle-specific inhibition of Mstn signaling confers protection against high-fat diet-induced insulin resistance [60,131]. Notably, even in severe diabetic models, such as the lipodystrophic A-ZIP/F-1 mice, muscle-specific Mstn inhibition ameliorates hyperglycemia and hyperinsulinemia, indicating that therapeutic efficacy functions independently of adipose tissue mass [60].

Mechanically, Mstn disrupts skeletal muscle glucose homeostasis by simultaneously suppressing the IRS-1/PI3K/Akt axis and the AMPK-PGC-1α pathway. These molecular perturbations impair insulin-stimulated GLUT4 translocation and mitochondrial bioenergetics [122].

The therapeutic potential of targeting the Mstn pathway is substantiated by a Phase 2 trial of bimagrumab (a monoclonal antibody targeting ActRIIA/B receptors) in patients with type 2 diabetes. Over 48 weeks, treatment elicited significant metabolic benefits, characterized by a placebo-adjusted HbA1c reduction of 0.72%. Crucially, this glycemic improvement coincided with substantial body composition remodeling, comprising a ~3.6 kg gain in lean mass and a concomitant 3.4 kg reduction in total body fat [59].

#### 3.2.12. Metabolic Dysfunction-Associated Steatotic Liver Disease (MASLD)

MASLD, formerly known as non-alcoholic fatty liver disease (NAFLD) [132], is characterized by the presence of hepatic steatosis and at least one of five cardiometabolic risk factors (increased body mass index (BMI), hypertension, diabetes mellitus, dyslipidemia, or hypertriglyceridemia) [133,134,135]. The natural course of MASLD typically progresses through distinct stages, including simple hepatic steatosis (early stage), non-alcoholic steatohepatitis (NASH, moderate stage), hepatic fibrosis and ultimately hepatic cirrhosis, which is the end-stage of the disease [136]. In early-to-moderate MASLD, pro-inflammatory cytokines (e.g., TNF-α and IL-6) activate the NF-κB pathway to upregulate intramuscular Mstn, thereby suppressing satellite cell myogenesis and promoting proteolysis. As the disease advances to cirrhosis, Mstn expression is further exacerbated by hyperammonemia. This metabolic hallmark of end-stage liver disease drives transcriptional Mstn upregulation via NF-κB, contributing to the severe sarcopenia characteristic of decompensated cirrhosis [137].

Clinically, elevated serum and intramuscular Mstn levels correlate strongly with sarcopenia severity and reduced survival in cirrhotic patients [138]. ActRII inhibitors and neutralizing antibodies against mature Mstn have shown promise in preclinical and early clinical studies by increasing muscle mass and improving metabolic parameters [109]. However, their effects on hepatic lipid metabolism and fibrosis in patients with concurrent sarcopenia and MASLD (including cirrhosis) remain unevaluated systematically. Adjunctive approaches include ammonia-lowering agents, such as rifaximin, which have been shown to reduce Mstn protein expression and restore muscle proteostasis in animal models [139]. Regular resistance exercise has also been shown to increase muscle mass and improve muscle function in patients with cirrhosis, partly through Mstn suppression [140].

## 4. Mstn-Related Pathway Targeted Drugs

**Figure 4:**
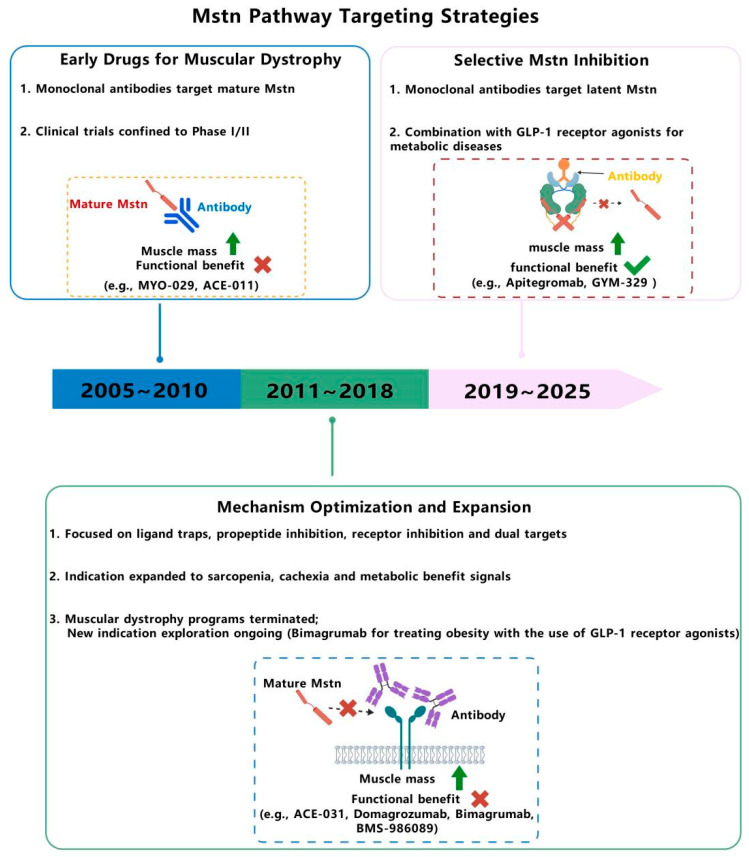
**Timeline of Mstn pathway targeting strategies (2005–2025):** (1) 2005~2010: Early muscular dystrophy drugs (monoclonal antibodies targeting mature Mstn), limited to Phase I/II trials (e.g., MYO-029 and ACE-011). (2) 2011~2018: Mechanism optimization (ligand traps and dual targets) and indication expansion (sarcopenia and cachexia); muscular dystrophy programs (e.g., ACE-031, domagrozumab, bimagrumab (a monoclonal antibody targeting ActRIIA/B receptors), and BMS-986089) terminated, with new obesity-focused trials (bimagrumab + GLP-1 receptor agonists). (3) 2019~2025: Selective latent Mstn inhibition (e.g., apitegromab and GYM-329) with ongoing combination therapy in metabolic disease (Mstn + GLP-1 receptor agonists).

**Figure 5:**
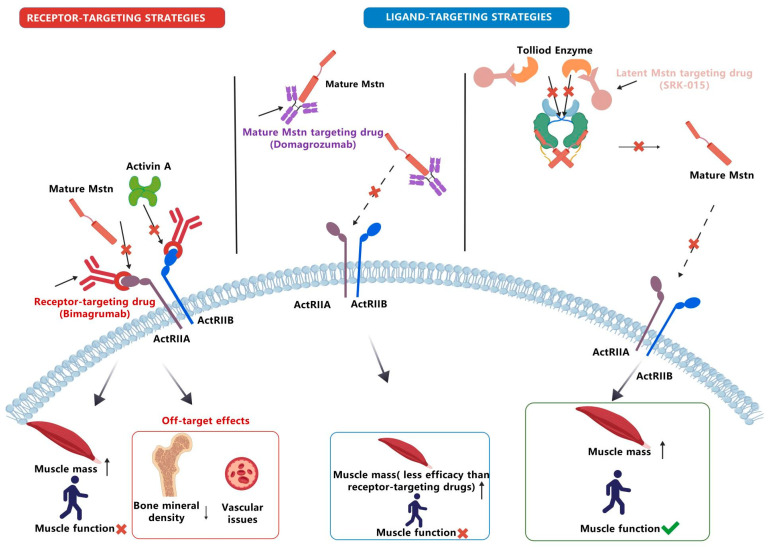
**Conceptual model of signaling network interactions and translational challenges in Mstn-targeted therapies.** This figure illustrates why distinct therapeutic strategies encounter specific translational bottlenecks. (1) Receptor-targeting drugs (e.g., bimagrumab) bind ActRIIA/B receptors to inhibit the binding of mature Mstn and Activin A. This approach increases muscle mass, but it does not improve muscle function and causes off-target effects like reduced bone mineral density and vascular issues. (2) Ligand-targeting drugs (mature form targeting) (e.g., domagrozumab) bind mature Mstn to boost muscle mass, though this effect is weaker than that of receptor-targeting drugs, and it also fails to enhance muscle function. (3) Ligand-targeting drugs (latent form targeting) (e.g., SRK-015) inhibit Tolloid enzyme-mediated processing of latent Mstn into its mature form, which allows this strategy to both increase muscle mass and improve muscle function. This figure contrasts these network-level failures with emerging combination or upstream strategies, providing a visual framework for the paradigm shift required in future drug development.

Current therapeutic strategies targeting the Mstn pathway are primarily divided into two categories: receptor-targeting (indirect Mstn-targeted) and ligand-targeting (direct Mstn-targeted) approaches (Figure 4 and Figure 5).

### 4.1. Direct Mstn-Targeted Therapeutic Strategies

Therapeutic strategies for direct Mstn neutralization primarily utilize monoclonal antibodies to inhibit ligand activity. Although the pioneering agent MYO-029 establishes the clinical safety of this approach, it fails to demonstrate significant functional benefits. This challenge of translating target engagement into clinical efficacy persists, with subsequent candidates such as domagrozumab and trevogrumab also showing limited functional improvement. Newer therapeutics aim for greater precision by targeting the precursor or latent forms of Mstn, as seen with SRK-015 and GYM-329, offering renewed potential for specific neuromuscular disorders (Table 1).

### 4.2. Indirect Mstn-Targeted Therapeutic Approaches

Drugs targeting activin receptors (ActRIIA/IIB), including ligand traps like ACE-031 and antibodies like bimagrumab, act by broadly blocking the interaction between ActRIIA/IIB and multiple TGF-β ligands, such as Mstn and activins. While they potently increase muscle mass and bone density, their clinical translation is hampered by significant limitations. A key challenge is the frequent dissociation between mass gains and functional improvement. More critically, broad receptor inhibition leads to off-target toxicities. These limitations underscore the need for more selective targeting strategies to improve the therapeutic window (Table 2).

## 5. Challenges and Future Perspectives

Chronic neuromuscular junction (NMJ) dysfunction represents a critical bottleneck in the translation of Mstn inhibition into functional recovery. While Mstn suppression reliably induces muscle hypertrophy, both clinical and preclinical data reveal a frequent uncoupling of muscle mass from functional strength. In spinal muscular atrophy (SMA), SMN deficiency precipitates motor neuron degeneration and NMJ structural fragmentation, characterized by disrupted acetylcholine receptor (AChR) organization and reduced MuSK expression. These deficits compromise the synaptic “safety factor,” rendering the neuromuscular system unable to effectively actuate hypertrophic muscle fibers. Notably, non-specific Mstn inhibition, even in the context of robust muscle growth, fails to reverse NMJ structural or functional deficits in preclinical models of neuromuscular disease, leaving hypertrophied muscle fibers functionally disconnected from motor neuron input.

However, the therapeutic landscape evolves, driven by emerging evidence that selective inhibition of latent Mstn facilitates superior functional preservation compared to inhibitors targeting mature Mstn or its receptors. Previous strategies targeting mature Mstn or the Activin type II receptor (ActRII receptor) primarily induce muscle hypertrophy but fail to prevent progressive functional deterioration in affected populations. This limitation is closely associated with the efficacy-safety trade-offs inherent to agents targeting the Mstn-ActRII pathway. In contrast, SRK-015, a monoclonal antibody targeting latent Mstn, demonstrates a distinct clinical profile. Data from the Phase 2 TOPAZ trial underscore this divergence. Over a 12-month period, non-ambulatory SMA patients treated with SRK-015 exhibit stabilization of motor function, as evidenced by the maintenance of Hammersmith Functional Motor Scale Expanded (HFMSE) scores. This outcome differs markedly from the natural history of SMA, wherein non-ambulatory patients typically experience a progressive annual decline in HFMSE scores [81]. The above data highlights the untapped potential of upstream targeting strategies.

This capacity to decouple muscle preservation from functional decay implies that targeting the latent form of Mstn may protect neuromuscular integrity. Although direct histological evidence linking SRK-015 to improved NMJ morphology remains to be elucidated, the observed clinical stabilization suggests that highly specific inhibition of latent Mstn may avoid the deleterious off-target effects on TGF-β family members essential for synaptic maintenance, thereby allowing the neuromuscular system to sustain function. Building on this premise, targeting pro-Mstn could offer even greater advantages. By intercepting Mstn activation at the earliest stage of biosynthesis, pro-Mstn targeting may maximize specificity, minimizing residual signaling that could compromise NMJ structure and ensuring that therapeutic interventions robustly support neuromuscular functional integrity rather than merely increasing mass.

To fully bridge the gap between muscle mass and motor performance, a multimodal paradigm is imperative. Future research should prioritize the strict evaluation of neuromuscular integrity by combining immunohistochemical analysis of end-plate structure with electrophysiological assessments, particularly when testing early-stage Mstn inhibitors. Additionally, maximizing therapeutic efficacy will probably require the combination of pro-Mstn or latent Mstn inhibition with NMJ-stabilizing agents, neuromuscular adaptation training, and nutritional support (such as essential amino acids) to promote contractile protein turnover [166]. Ultimately, shifting the focus from mature Mstn inhibition to early-stage Mstn inhibition offers a promising avenue to overcome the synaptic barriers in SMA and redefine the efficacy of Mstn-targeted therapies.

The current landscape of drug candidates targeting the Mstn-ActRII pathway reveals a complex trade-off between efficacy and safety, further complicated by the functional divergence between Mstn and its close homolog, GDF11. A major factor limiting the clinical translation of Mstn-targeted therapies is the profound signaling redundancy within the TGF-β superfamily. While Mstn is the primary negative regulator of skeletal muscle mass, GDF11 shares approximately 90% sequence identity and utilizes overlapping receptors (ActRIIA/B), yet it appears to exert distinct physiological roles [1]. Unlike Mstn, which is predominantly myogenic, GDF11 functions as a systemic regulator with reported positive effects on mitochondrial biogenesis, insulin sensitivity, and skeletal muscle oxidative capacity [167]. This functional dichotomy has critical therapeutic implications. In pathological microenvironments, such as DMD, cancer cachexia, and sarcopenia, chronic inflammation robustly upregulates Activin A, creating a biological bypass that blunts the efficacy of specific Mstn inhibitors. Conversely, broad-spectrum receptor blockade (e.g., ACE-031, bimagrumab) attempts to overcome this redundancy but inadvertently neutralizes GDF11 along with Mstn and Activin A. Because GDF11 signaling supports metabolic homeostasis and mitochondrial integrity, its unintended inhibition may deprive muscle tissue of essential metabolic support. This mechanistic insight helps explain the recurring clinical observation of mass-function mismatch: while broad ActRII blockade induces robust hypertrophy, the concurrent loss of GDF11-mediated metabolic signaling may result in muscle that is larger but metabolically inefficient and fatigable. Furthermore, broad blockade disrupts Activin A and BMP9 signaling essential for vascular stability, leading to severe adverse events like telangiectasias and epistaxis, which ultimately halted the development of ACE-031 despite its efficacy in increasing lean mass [152]. Therefore, the therapeutic bottleneck is defined by a paradox: ligand-specific strategies lack clinical efficacy due to redundancy bypass, while receptor-level strategies compromise functional outcomes and safety due to the loss of beneficial GDF11 and Activin A signaling. This underscores the urgent need for next-generation therapeutics, such as ligand-specific traps, tissue-targeted delivery systems, or aptamers, that can selectively dissect Mstn signaling from Activin A/GDF11 pathways [168,169,170].

Conversely, Mstn-targeting candidates, such as domagrozumab (which targets mature Mstn), demonstrate a lack of efficacy in key functional outcomes despite promoting muscle growth [147]. The mechanisms of muscle growth differ fundamentally between species. The iconic “double-muscling” phenotype observed in Mstn-null mice is largely attributed to developmental hyperplasia, an increase in the number of muscle fibers formed during embryogenesis [1]. This developmental plasticity allows for dramatic increases in muscle mass. However, this mechanism is biologically inaccessible in adult human patients. In contrast to the developmental plasticity of rodents, adult human skeletal muscle possesses a fixed myofiber number and is strictly limited to hypertrophy (an increase in the size of existing fibers). Consequently, the magnitude of mass gain achievable in mice sets an unrealistic therapeutic ceiling for adult humans, where clinical gains are typically modest [77,147].

Notably, the efficacy gap in Mstn inhibitors is particularly prominent in sarcopenia, the primary indication for muscle-wasting therapies, where no conclusive evidence currently supports the successful therapeutic application of Mstn inhibitors. Instead, the focus of preclinical and clinical investigation shifts toward models of muscle loss induced by GLP-1 receptor agonists. This strategic pivot highlights two core issues. Firstly, sarcopenia is a multifactorial syndrome characterized not only by reduced muscle mass but also impaired neuromuscular function, chronic inflammation, metabolic disorders, and blunted anabolic sensitivity. Mstn inhibitors, which primarily target myogenic pathways, fail to address the complex pathological network, leading to poor translation of muscle mass gain into functional improvements. Secondly, the widespread clinical application of GLP-1 receptor agonists for obesity and T2DM has drawn attention to their side effect of inducing skeletal muscle loss. Unlike sarcopenia, GLP-1 receptor agonist-induced muscle atrophy involves a simpler pathological mechanism primarily centered on energy metabolism and protein synthesis. Consequently, Mstn inhibitors directly counteract this catabolic drive more effectively, demonstrating more pronounced therapeutic effects.

However, the therapeutic efficacy demonstrated in GLP-1-induced models does not necessarily translate to the multifactorial pathology of sarcopenia. In the current landscape of GLP-1 therapeutics, the approach to the ActRII axis evolves to prioritize preservation or enhancement of lean body mass alongside weight loss induced by metabolic agents. The strategic goal is to align weight loss trajectories with functional improvements, thereby preventing divergence between these physiological outcomes. Achieving this alignment requires a comprehensive reconfiguration of clinical trial design. This includes carefully considering baseline population characteristics, understanding exposure-effect temporal dynamics, and developing strategies for drug discontinuation and follow-up.

In addition to critical limitations, such as the off-target toxicity associated with receptor-targeting agents, the insufficient functional efficacy of mature Mstn-directed therapies in sarcopenia, and the poor clinical translatability of GLP-1-induced muscle loss models, antibody-based Mstn therapeutics encounter another significant barrier that compromises their clinical potential, specifically their limited ability to penetrate muscle tissue [168].

As Mstn exists extracellularly as an uncleaved pro-protein within the muscle microenvironment, the poor permeability of large antibodies limits their ability to effectively neutralize this target [168]. To overcome this limitation, the development of molecules with superior muscle tissue penetration presents a compelling alternative, such as aptamer-based therapy [169,170]. By leveraging the specific mechanism of Mstn activation, these molecules can be designed to bind to functional domains with high affinity, such as the receptor-binding interface or, most notably, the Furin cleavage motif within the pro-domain. Targeting the Furin recognition site intercepts the activation cascade at its absolute inception, preventing latent complex formation. Crucially, because it masks a unique sequence on the Mstn substrate rather than blocking shared receptors, this strategy elegantly bypasses both the systemic toxicity of pan-receptor traps (e.g., BMP9 disruption) and the “redundancy trap” of mature Mstn antibodies (e.g., Activin A bypass), thereby avoiding the off-target effects inherent to non-selective inhibitors. In addition, future application of single-cell multi-omics will be critical for mapping Mstn signaling heterogeneity within the muscle microenvironment, potentially identifying specific fibro-adipogenic or immune cell subpopulations that drive resistance to Mstn inhibition [171].

In summary, bridging the gap between muscle mass accretion and functional recovery requires a paradigm shift in Mstn-targeted therapies. The development of highly specific molecules with enhanced muscle permeability to intercept Mstn maturation represents a promising avenue for safer interventions. Coupled with the growing recognition that Mstn functions diverge significantly across adipose, bone, and immune microenvironments, it is clear that systemic receptor blockade is no longer viable. Instead, next-generation therapeutics must evolve toward tissue-specific delivery, rational combination regimens, and upstream pro-domain targeting to navigate multifactorial pathology while preserving systemic homeostasis.

## Figures and Tables

**Table 1:** Clinical studies exploring Mstn inhibition strategies (direct Mstn-targeted therapeutic approaches).

Name	Type	Target/Molecule	Target Indication	Primary Therapeutic Effect	Safety/Clinical Status	Key Features/Notes
GYM-329 [141,142]	Targeting latent Mstn monoclonal antibody	Specific epitope on latent Mstn	Spinal muscular atrophy (SMA) (Target population: 2–10 years old, ambulatory SMA patients receiving Risdiplam)	1. Improved patient compliance with subcutaneous administration. 2. Potential synergy with Risdiplam in muscle function repair. 3. Optimized efficacy for Risdiplam-treated SMA patients.	1. Mid-term data (abstract) disclosed; final clinical data pending publication. 2. Limited to patients aged 2–10 years receiving Risdiplam. 3. Long-term metabolic safety remains to be established. The ongoing Phase 3 trial (NCT05115110) focuses on safety, pharmacokinetics, and pharmacodynamics as primary endpoints. Parallel preclinical studies are evaluating the potential synergy with exercise to enhance endurance in aged models.	Binds to latent Mstn, blocking cleavage by BMP-1/TLD protease, inhibiting activation and release of mature Mstn.
MYO-029 (Stamulumab) [143,144]	Anti-Mstn monoclonal antibody (IgG1)	Mature Mstn	DMD	Muscle mass and function improvement not shown.	Stop development (Wyeth advancing to Pfizer).	Early anti-Mstn monoclonal antibodies represent the first clinical validation of the limitations of monoclonal antibody strategies.
Landogrozumab (LY2495655) [145,146]	Anti-Mstn monoclonal antibody	Mature Mstn	Disuse muscle atrophy, cancer cachexia (pancreatic cancer)	Muscle mass improvement is weak or non-existent.	Cancer patients experience a high incidence of severe adverse events, leading to the discontinuation of development (Eli Lilly).	Safety issues are prominent in cachexia indications, limiting its application.
Domagrozumab (PF-06252616) [49,147]	Anti-Mstn monoclonal antibody	Mature Mstn	DMD, LGMD2L	Muscle mass improvement was limited and did not meet the primary endpoint.	Development termination (Pfizer).	For genetic myopathies, there has been no breakthrough in efficacy beyond the early monoclonal antibody bottleneck.
Trevogrumab (REGN1033) [148]	Anti-Mstn monoclonal antibody	Mature Mstn (almost moderate cross-reactivity with GDF11)	Sarcopenia and inclusion body myositis (IBM)	Did not achieve the expected therapeutic effect.	Terminate development (including joint Garetosmab trial with Regeneron).	Target specificity optimization (reducing cross-reactivity with GDF11, but moderate cross-binding to GDF11 remains), and functional improvement was not achieved in clinical trials.
SRK-015 [81,149,150]	Monoclonal antibodies targeting the latent complex of Mstn (LAP/pro-domain)	Mstn latent complex (induces conformational changes, preventing its activation and release)	SMA, muscular atrophy/bone-related diseases	Improving muscle mass and function, improving muscle mass/function (clinical development), and promoting bone growth (preclinical, SMA mice).	Currently still under development.	Targeting latent Mstn that binds to the extracellular matrix, with localized action advantages; currently the most promising immune neutralizing agent.
BMS-986089/RG6206 [151]	Adnectin(fusion protein based on the Fn3 structural domain)	Mstn + GDF11	DMD	Did not achieve the expected therapeutic effect.	The DMD trial failed, and the project was terminated (Roche).	Fusion proteins based on non-antibody scaffolds targeting Mstn and GDF11 have not been successful in clinical trials.

**Table 2:** Clinical studies exploring Mstn inhibition strategies (indirect Mstn-targeted therapeutic approaches).

Name	Type	Target/Molecule	Target Indication	Primary Therapeutic Effect	Safety/Clinical Status	Key Features/Notes
ACE-031 [152,153]	Soluble human/mouse ActRIIb ECD-Fc fusion protein	Mstn, Activin, GDF11	DMD, ALS	Significantly increases muscle mass, bone density, and improves muscle strength; effective in the model.	A clinical trial for DMD boys was halted due to symptoms similar to HHT (nosebleeds, mucosal bleeding); it is speculated to be related to interference with BMP9 signaling.	One of the earliest ligand-capture drugs to gain significant attention.
ACE-083 [154,155]	Modified truncated Follistatin (FS291)-Fc fusion protein	Mstn, Activin (via Follistatin sequestration, local muscle delivery)	Facial or limb muscle atrophy (such as facioscapulohumeral muscular dystrophy)	Did not significantly improve muscle function or quality of life.	The development has been suspended.	Improved affinity for the extracellular matrix, reduced systemic distribution, theoretically decreasing the impact on off-target tissues such as vascular endothelium.
ACE-536 (Luspatercept) [156]	ActRIIb extracellular domain (residues 24–131)-Fc fusion protein (structurally modified)	Decrease affinity for BMP9, GDF11, and Mstn, while retaining activity on some members of the TGF-β superfamily.	Chronic anemia (Mediterranean anemia, myelodysplastic syndromes)	Improve chronic anemia.	Approved by the FDA.	Non-muscle adaptation drugs, but validating the efficacy of ligand capture strategies in blood diseases.
ACE2494 [157]	Further optimized ActRIIb extracellular domain (ECD)	Mstn, GDF11	Not specified (suspected muscle/bone-related)	ACE-2494: Did not meet expectations.	The development of ACE-2494 has been paused due to the production of drug-resistant antibodies by the subjects.	Subsequent optimized versions of ActRIIbECD, improving safety or target specificity.
Bimagrumab (BYM338) [59,125,158,159]	Fully human IgG1 (targeting ActRIIa and ActRIIb, with higher affinity for ActRIIb)	ActRIIa, ActRIIb (block ligand binding)	IBM, sarcopenia, cachexia	Significantly increases muscle mass, but functional improvement is not obvious; reduces fat and improves insulin resistance (diabetes model).	1355 participants, 11 trials (including 3 Phase III); IBM trials did not meet functional endpoints and have been removed from the pipeline (Novartis).	Simultaneously inhibiting both ActRII subtypes significantly increases muscle mass but lacks functional benefits, which is the key failure point.
AAV1:FS344 [160]	AAV vector-mediated expression construct of Follistatin-344	Mstn, Activin	Inclusion body myositis (IBM), DMD	Muscle structure improvement, increased six-minute walk distance, and other functional indicators improved.	The sample size is small, with a focus on safety studies; no impact on reproductive hormones (FS315 does not bind to heparin/HS), Duchenne muscular dystrophy (DMD), and inclusion body myositis (IBM), Phase I/II (NCT03362502). The AAV vector shows superior muscle targeting, with sustained Follistatin expression, and muscle strength and safety indicators are controllable.	Local delivery avoids FS315 non-specific binding; AAV vector enables sustained muscle targeting; superior muscle strength/safety indicators in IBM.
LAE102 [161]	Monoclonal Antibody	Activin Receptor Type IIA (ActRIIA)	Muscle-wasting disorders	Increases lean mass, reduces fat mass (“quality weight loss”).	Phase I SAD completed (January 2025): Favorable profile, no SAEs. Phase I MAD ongoing. U.S. collaboration with EliLilly.	Selectivity for ActRIIA over ActRIIB has the potential to minimize off-target risks (e.g., diarrhea); Shows synergy with GLP-1 receptor agonists.
ALG-801 [162]	Ligand Trap (ActRIIA/IIB Hybrid)	Mstn (GDF8), Activin, GDF11	Pulmonary arterial hypertension (PAH), neurodegenerative diseases	Inhibits Activin/Mstn/GDF11 (TGF-β superfamily branch) signaling; addresses vascular remodeling and protein aggregation.	Phase 1a/1b completed. FDA Orphan Drug Designation for PAH (January 2025). Phase 2 trials planned.	Orally bioavailable, a significant advantage over traditional biologics. Positioned as a platform therapy for multiple diseases.
HS235 [163,164]	Antibody-Fusion Protein	GDF/Activin receptors	Pulmonary arterial hypertension (PAH)	Aims to restore balanced TGF-β signaling to reduce pulmonary resistance and remodel adipose tissue.	Phase I trials ongoing; preliminary data (2025) show robust target engagement and favorable safety profile.	Oral bioavailability.
KER-065 [165]	Ligand Trap (ACVR2A/ACVR2B-Fc Fusion)	Activin A, Mstn	Duchenne muscular dystrophy (DMD)	Promotes muscle regeneration; reduces fibrosis; increases bone mineral density.	Phase 1 completed: Favorable safety profile. Phase 2 ongoing for DMD. FDA Orphan Drug Designation (Aug 2025).	Dual mechanism targeting multiple pathways (Mstn and Activin).
GenSci156 [126]	Bispecific antibody	Activin II receptors (ActRIIA/B) and Glucose-dependent insulinotropic polypeptide receptor (GIPR)	Obesity	Efficient fat reduction while increasing lean body mass; improves glucose metabolism and insulin sensitivity; no obvious impact on appetite.	Preclinical stage; no human safety data available yet; preclinical studies show good efficacy without obvious adverse effects related to appetite suppression.	Developed by Jinsai Pharmaceutical. Aopts dual-target synergy to block Mstn-mediated metabolic disorders and regulate glucose metabolism. Subverts the disadvantage of traditional weight-loss drugs that cause muscle loss.

These figures are entirely original work created by the authors for this manuscript. It was generated using BioGDP.com (https://biogdp.com/) and has not been reused or adapted from any previously published work.

## Data Availability

No new data were created or analyzed in this study. Data sharing is not applicable to this article.
